# Fusions of Tumor-derived Endothelial Cells with Dendritic Cells Induces Antitumor Immunity

**DOI:** 10.1038/srep46544

**Published:** 2017-04-24

**Authors:** Yingying Huang, Qiqi Mao, Jian He, Jing Su, Yi Peng, Wei Liang, Zixi Hu, Sufang Zhou, Xiaoling Lu, Yongxiang Zhao

**Affiliations:** 1 National Center for International Research of Biological Targeting Diagnosis and Therapy, Guangxi Key Laboratory of Biological Targeting Diagnosis and Therapy Research, Collaborative Innovation Center for Targeting Tumor Diagnosis and Therapy, Guangxi Medical University, Shuang Yong Rd. 22, Nanning 530021, P. R. China

## Abstract

To explore dendritic cells/tumor-derived endothelial cells (DC/EC) fusion cells are potent stimulators of T cells to impact tumor progression. ECs were isolated from mice hepatoma cell line (H22) Xenograft, and dendritic cells were isolated from bone marrow of BALB/c mice, then the isolated ECs were cultured and detected the endothelial surface expression of CD105 by flow cytometry. The endothelial characteristics of ECs were detected by tube formation assay and Dil-Ac-LDL uptake assay. After the fusion with polyethylene glycol (PEG), we used DCs, ECs, DCs mixed ECs as the control groups, DC/EC fusion cells as the experimental group, Secretion of IFN-α and IFN-γ was evaluated, T lymphocyte proliferation and cytotoxic T lymphocytes (CTL) were detected *in vitro. In vivo*, T lymphocyte induced by five groups was injected to detect the effect of tumor progression. Purified ECs (CD105^+^) took the function of endothelial cells, then successfully fused with DCs. The DC/EC fusion cells were functional in stimulating the proliferation of T cells, which produced IFN-α and IFN-γ. *In vivo*, T cells stimulated by DC/EC fusion cells effectively repressed tumor growth. The fusion cells, which was capable of stimulating T cells, is indispensable for antitumor immunity.

Dendritic Cells (DCs) are regarded as antigen-presenting cells, which has the capacity of eliciting primary immune responses^1^,^2^. DCs collect and dispose the antigens into peptides, then DCs present peptides in MHC classes I and II and costimulatory molecules for identification by T cells^1^,^3^,^4^.

Angiogenesis plays a significant role in promoting tumor progression. Tumor development beyond 1–2 mm is dependent on the formation of a functional blood supply system for nutrient delivery. Based on previous studies involving established cell lines or vessels, the blood vessels of tumor and those of normal tissues differ in permeability, composition of the basement membrane, extracellular matrix and cellular composition^5^,^6^,^7^. When compared to normal blood vessels, tumor vessels are tortuous, poor organizational characteristics, high permeability and inclined to leaky take the macromolecules of tumor microenvironment to blood circulation^8^,^9^. Normal vascular endothelial cells derived from embryos, while about 50% to 60% of tumor vascular endothelial cells derived from tumor stem cells, coexpress the specific antigens of tumor cells^10^,^11^. Tumor vascular endothelial cells can split proliferation continuously, however normal vascular endothelial cells could not^12^. CD105 has been suggested to be the most suitable marker available to quantify tumor angiogenesis, which was observed in tissues undergoing active angiogenesis, whereas absent in blood vessels within normal tissues^13^. In this study, the tumor vascular endothelial cells we selected all express CD105. Thus, it plays an essential role of anti-cancer therapy by targeting tumor endothelial cells.

Previously strategy is the fusion of DCs and tumor cells^14^,^15^,^16^. In this approach, tumor-derived endothelial Ags are delivered to DCs. Such as APCs, the fusion cells (DC/EC) function with the ability to migrate to draining lymph nodes, where they interact with T cells and induce effective antitumor immunity^17^,^18^,^19^. These results make clear that tumor-derived endothelial Ag presentation targeting activation of T cells is necessary in antitumor immunity.

## Results

### Purification and characterization of CD105^+^ cells

**Figure 1:**
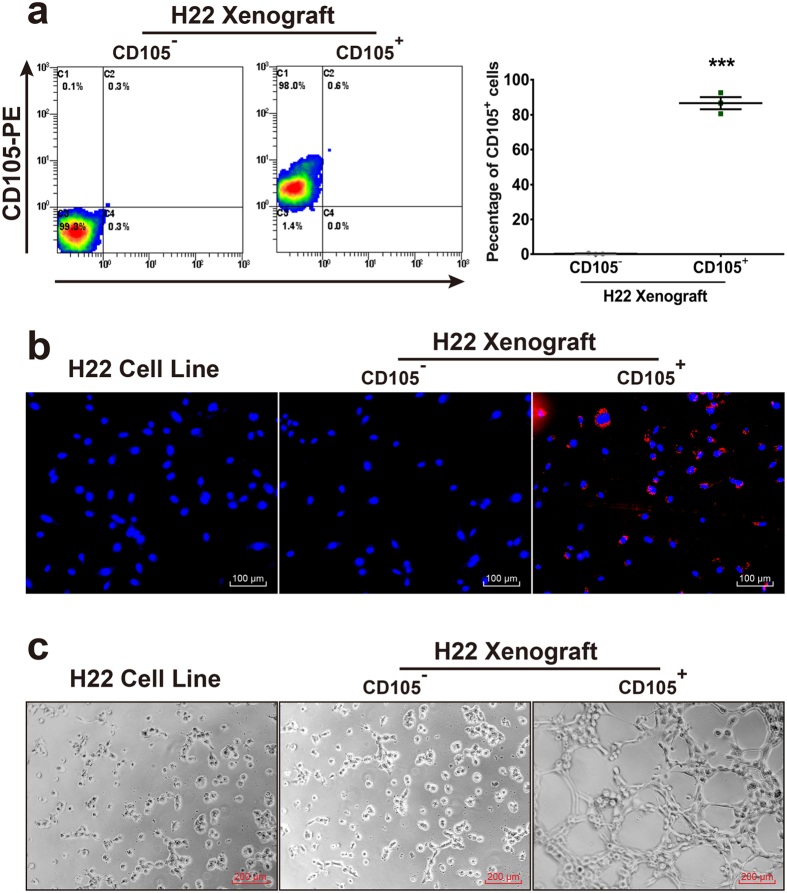
Purification and characterization of CD105^+^ cells. (**a**) Flow cytometry plot data obtained using CD105^+^ antibody to quantify endothelial cells. (**b**) CD105^+^ cells take up acetylated LDL compared to CD105^−^ cells and H22 cell lines. (**c**) CD105^+^ cells form capillary-like networks on Matrigel compared to CD105^−^ cells and H22 cell lines. Scale bars = 100 μm in (**b**), =200 μm in c. ***P < 0.001.

CD105^+^ cells were purified from H22 Xenografts by magnetic activated cell sorting (MACS)^20^, CD105^+^ and CD105^−^ cells were separated from the single cell suspensions and analyzed the expression of CD105 by flow cytometry. The fractions of CD105 expression cells in CD105^+^ cells or CD105^−^ cells of tumor Xenograft were 97.6 ± 1.4% and 0.3 ± 0.1%. These results indicate an excellent enrichment of CD105^+^ subpopulations by the magnetic cell separation (Fig. 1a). To compare the endothelial cell function of CD105^+^ cells with that of CD105^−^ cells, tube formation assay and Dil-Ac-LDL uptake assay were detected. In Fig. 1b and c, CD105^+^ cells from tumor tissue showed Dil-Ac-LDL up-take and formation of endothelial tubes, respectively. These results suggested that CD105^+^ cells took of endothelial cell function.

### Characterization of the DC/EC fusion cells

**Figure 2:**
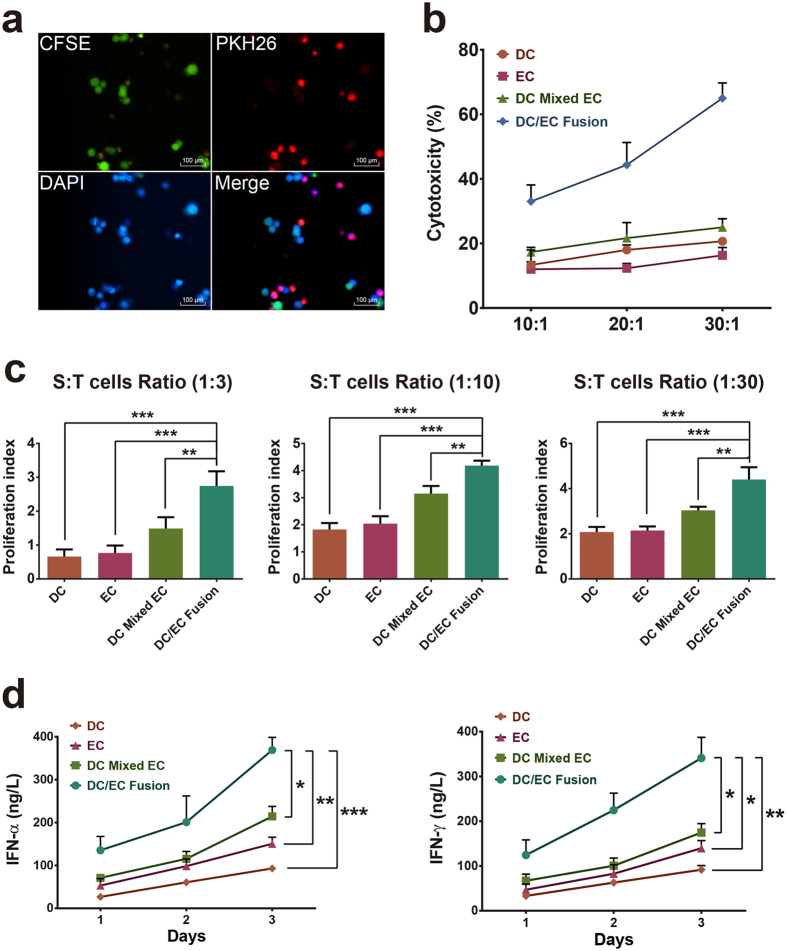
Phenotype of DC/EC and stimulation of antitumor CTL cells by DC/EC. (**a**) ECs were stained with CFSE (Green), DCs were stained with PKH26 (Red) and the nuclear were stained with DAPI (Blue). Scale bars = 100 μm. (**b**) Cultured with DC only, EC only, DC mixed with EC, or DC/EC fusion, then the stimulated T cells were incubated with PKH-26-labled target ECs at the indicated effector-to-target cell (10:1, 20:1, 30:1), and then detected for lysis. (**c**) The proliferation of T cells was measured after cultured with DC only, EC only, DC mixed with EC, or DC/EC fusion. (**d**) The IFN-α and IFN-γ production of T cells were analyzed by ELISA. *P < 0.05, **P < 0.01, ***P < 0.001.

Immunofluorescence was used to assess efficiency of the fusions. In Fig. 2a, endothelial cells dyed CFSE, DCs dyed PKH26 and the fusion cells (DC/EC) expressed both of fluorescence. These findings demonstrate the formation of heterokaryons by fusing endothelial cells to DC successfully.

### Induction of endothelial/Fusion Cells (FCs)-specific CTL responses by four types of cell preparations

To investigate the Ag-specific CTL induction capacity by four types of cell preparations, cytotoxicity assays were performed. Incubation of the endothelial/FCs-stimulated T cells demonstrated selective lysis of the tumor endothelium, while there was no significant lysis of the DC only, EC only, DC mixed with EC (Fig. 2b). These results indicate that T cells stimulated by DC/EC fusion cells had selective lysis of tumor endothelium.

### Stimulation of T cell proliferation compromised in fusion cells

To determine the ability of fusion cells about stimulating T cell proliferation, flow cytometry (Beckman Coulter Epics XL, USA) was applied. T cells co-cultured with DC/EC fusion cells proliferated vigorously, co-cultured of these T cells with DC only, EC only, DC mixed with EC resulted in proliferation of T cells, however, at a lower level (Fig. 2c).

### IFN-α and IFN-γ production of T cells by four types of cell preparations

To compare the activation of T cells by four types of cell preparations, we detected IFN-α and IFN-γ production by ELISA. The group of DC/EC fusion cells were superior in producing IFN-α and IFN-γ in T cells. In contrast, however, there was little, if any, IFN-α and IFN-γ production in T cells co-cultured with cell suspensions of DC only, EC only, DC mixed with EC (Fig. 2d).

### DC/EC fusion cells induce antitumor activity

**Figure 3:**
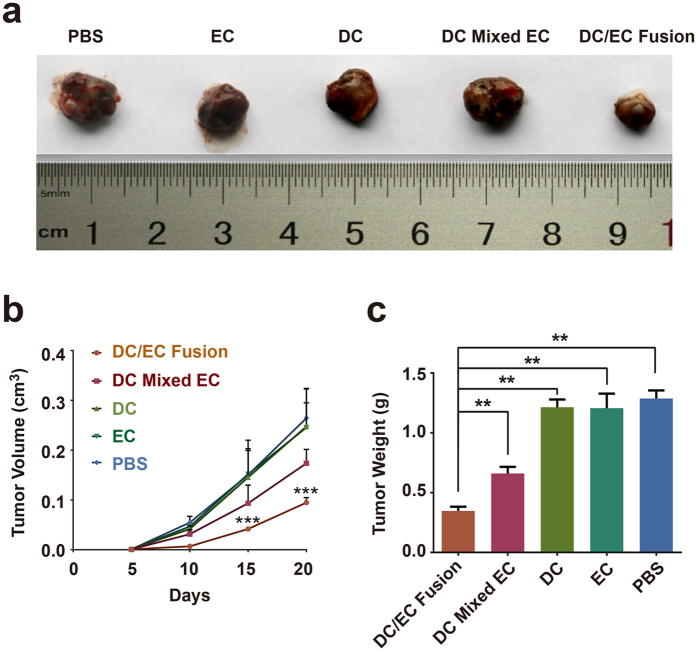
T cells activated by DC/EC fusion inhibit H22 Xenograft growth *in vivo*. (**a**) Photograph of tumor tissues dissected from mice. (**b**) Tumor volume measured every 5 days by caliper measurement up to 20 days. (**c**) Average Xenograft tumor weights at 20 days. n = 3. **P < 0.01, ***P < 0.001.

To assess the induction of antitumor immunity, mice were immunized with irradiated DC, EC, DC mixed with EC or DC/EC fusion cells and then challenged intravenously with 2 × 10^5^ viable H22 cells. In Fig. 3a, immunization of DC/EC fusion cells significantly decreased the tumorigenicity of H22 in BALB/c mice. The tumor volume and weight of the DC/EC fusion cells group was significantly decreased compare to the PBS group (Fig. 3b and c). These findings indicate that T cells induced by DC/EC fusion cells is effective in anti-tumor activity.

### Cell proliferation, endothelial cell expression and cell apoptosis in H22 Xenograft

**Figure 4:**
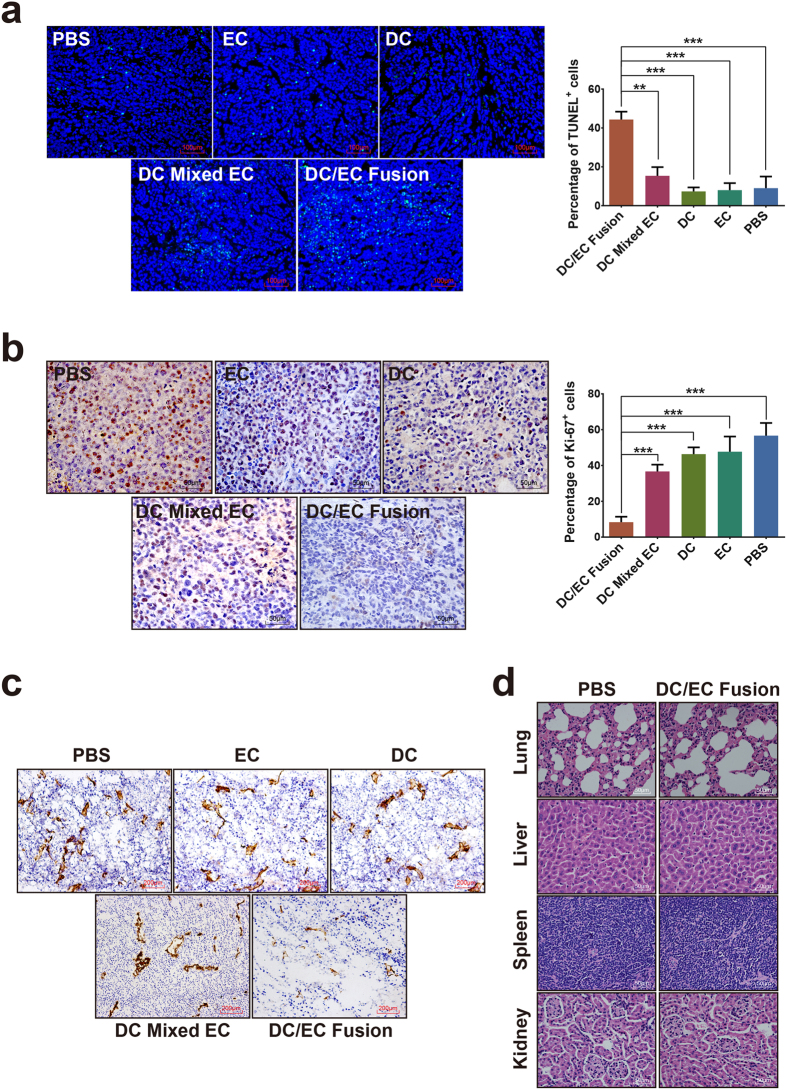
T cells activated by DC/EC fusion impact tumor apoptosis, proliferation and endothelial cell expression *in vivo*. (**a**) TUNEL expression in tumor tissues and quantitation of its expression. (**b**) Ki-67 expression in tumor tissues and quantitation of Ki-67 expression. (**c**) CD105 expression in tumor tissues. (**d**) HE staining of mice organs to determine toxicity of tissues. Scale bars = 100 μm in a, = 50 μm in b and d, = 200 μm in (**c**). **P < 0.01, ***P < 0.001.

In Fig. 4a, the result showed that there was a significantly increase in apoptosis in DC/EC fusion groups when compared to other groups. We explored the function of activated T cells by immunization, which was confirmed by IHC staining using Ki-67 and CD105 antibody (Fig. 4b and c). T cells induced by DC/EC fusion cells led to low level of Ki-67 protein and CD105 protein expression compared to that by mixed with DC and EC, DC only, EC only or PBS. Importantly, we found no toxicity in heart, lung, liver or kidney tissues of mice injected with T cells induced by immunization (Fig. 4d). These data indicate that T cells induced by DC/EC fusion cells can enhance antitumor immunity.

## Discussion

During the development of immune system, body can produce immune tolerance to autologous antigen, so in a healthy state, the body’s immune system will not produce an immune response to normal tissue. Previous study found that in situations where a tumor-associated antigen exists but remains unidentified, an approach may be needed for presentation of that antigen by a professional antigen-presenting cell (APC)^21^. Stimulus for DCs activation is in combination with Ags, mature DCs induce an effective anti-tumor immunity^22^,^23^,^24^,^25^,^26^,^27^. The DC/tumor fusion is an effective approach, because multiple tumor-associated Ags, including those known and unknown, are endogenously processed and presented by MHC class I and II pathways with the ability to migrate to draining lymph nodes^28^,^29^,^30^,^31^,^32^,^33^,^34^,^35^. In previous studies, DC-Tumor fusion have been found to have the effective treatment in carcinomas, lymphomas, and melanomas in mice^14^,^36^,^37^,^38^,^39^,^40^,^41^. These findings have recently been extended to the treatment and long-term survival of patients^14^,^42^,^43^,^44^,^45^. However, the fusion of DCs with tumor-derived endothelial cells and its function is largely unknown.

In this experimental setting, T cells are stimulated by four kinds of cell suspensions (DC only, EC only, DC mixed with EC, DC/EC fusion). The results demonstrate that T cells activated by DC/EC fusion cells are effective in protecting mice against tumor challenge. In this context, fusion of tumor-derived endothelial cells with DCs may result in heterokaryons that express the necessary MHC, DC/EC fusions as compared with mixed DC with EC, DC only or EC only, were sufficient to give rise to T cell proliferation, IFN-α, IFN-γ production and CTL responses. T cells treat neovascular antigens of tumor vascular endothelial cells as heterologous antigens (such as CD105). When these antigens are largely and stably presented to T cells by DCs, could activate the specific T cells, kill tumor vascular endothelial cells, but not target normal blood vessels^21^.

In conclusion, we have isolated and generated tumor-derived endothelial cells and DCs from tumor-bearing mice with success. Immunogenic cells created by DC/EC fusions have the capacity to greatly stimulate EC-specific T cells. Our findings that T cells activated by DCs fused with tumor-derived endothelial cells could induce higher levels of IFN-α and IFN-γ, as well as significantly reinforce CTL responses *in vitro* or *in vivo*, which may help in designing optimal strategies for the therapy and may improve DC/EC fusion-based vaccination strategies.

## Materials and Methods

### DC generation

DCs were obtained from bone marrow of BALB/c mice^17^. BALB/c mice were obtained from Vital River Company (Beijing, China) and were housed and cared for in accordance with the Federation of European Laboratory Animal Science Association guidelines, and all protocols were approved by the Animal Ethics Committee of Guangxi Medical University (Nanning, Guangxi, China). Briefly, bone marrow cells were flushed and cultured in RPMI 1640 medium supplemented with 20 ng/ml murine rGM-CSF (Sigma-Aldrich). After five days of culture, DCs were purified and harvested for fusion to endothelial cells.

### Preparation of endothelial cells

Tumors were removed from BALB/c mice bearing H22 and placed in cold PBS solution with 50 units/ml heparin. Peripheral and necrotic tissues were excised and remaining tumor was minced by using a scalpel. Dissociation of 0.1 × 0.1 × 0.1 cm^3^ minced tissue was performed in a 37 °C enzyme cocktail of 10 mg collagenase type I, 20 ml DMEM, 2 ml FBS for 60 min of constant mixing with vortex. The cell suspension was passed through 80 mesh strainer, PBS solution washed, then the cells were resuspended in 100 μl 0.01 M PBS buffer. Single cells were magnetically labeled with anti-CD105 Microbeads (Miltenyi Biotec) in the dark at 4 °C for 30 min and applied to the prepared MS Column (Miltenyi Biotec). CD105^+^ cells bound to the beads were flushed out by applying the plunger supplied with the column. Then, the sorted CD105^+^ cells were cultured by Endothelial Cell Medium (ScienCell).

### Flow Cytometry

For flow cytometry, cells were stained at the concentration of 1 × 10^6^ cells per 95 μl buffer and 5 μl phycoerythrin-conjugated anti-CD105 (ebioscience) at 4 °C for 30 min before flow cytometry analysis. All data were analyzed by EXPO32 Softwear.

### Tube Formation Assay

To analyze the tube formation ability of CD105^+^ cells, 100 μl/well of growth factor-reduced Matrigel (BD Bioscience) was laid into 96-well plates to solidify. Cells were seeded into 96-well plates. After 6 h, the tube formation was assessed with microscopy^20^.

### Dil-Ac-LDL Uptake Assay

CD105^+^ cells were plated into the 6-well plates at 5 × 10^4^ cells/dish. At 75% confluence, the culture medium was replaced by the serum-free DMEM for 24 h, followed by incubation with 2 μg/ml Dil-Ac-LDL for 5 h in incubator. Then cells were washed and fixed with paraformaldehyde (4 °C, 30 min), followed by DAPI staining for 3 min. The Dil-Ac-LDL uptake was assessed with microscopy^20^.

### Fusion of DCs with endothelial cells (DC/EC)

DCs were incubated with ECs for 5 min at a ratio of 10:1 in serum-free medium, which contained 50% polyethylene glycol. Then, culture medium was added to dilute the polyethylene glycol slowly. After washing, the cells were cultured in RPMI 1640 medium (10% FBS, 500 U/ml GM-CSF) for 7–14 days.

### Cell proliferation assay

The effects of T cells co-cultured with DC only, EC only, DC mixed with EC, or DC/EC fusion cells on cells proliferation was examined at stimulate cells : T cells (S : T cells) ratios by flow cytometry (Beckman Coulter Epics XL, USA).

### Cytotoxicity assays

T cells were stimulated and harvested, which were treated as effector cells in CTL assays^46^,^47^. The effects of T cells co-cultured with DC only, EC only, DC mixed with EC and DC/EC fusion cells for 5 hours, respectively. After T cells stimulation, 2 × 10^4^ PKH-26 (Sigma-Aldrich) labeled-target ECs were cultured with T cells (37 °C, 5 h). Cytotoxicity assays were examined at the indicated effector-to-target cell (E:T) ratios by flow cytometry (Beckman Coulter Epics XL, USA).

### ELISA

In order to assess the production of IFN-α or IFN-γ in T cells, DC only, EC only, DC mixed with EC, or DC/EC fusion cells were washed twice and cocultured with T cells (1 × 10^5^ cells) at a ratio of 1:10 in 48-well plates at 37 °C for 3 days in the absence of IL-2. T cells were purified and cultured in the low-dose of IL-2 (10 U/ml) for 3 days. The test supernatants from these samples were collected and tested for IFN-α and IFN-γ production by ELISA (BD Pharmingen) according to manufacturer’s instructions.

### Proliferation assay (*In vivo*)

BALB/c mice (Four weeks old, female) were purchased from Guangxi Laboratory Animal Center (Nanning, China). All animal care and experimental procedures were according to guidelines of the Institutional Animal Care and Use Committee of Guangxi Medical University. Left flanks of ten mice were implanted subcutaneously with 1 × 10^5^ H22 (mice hepatoma cell lines) suspended in 0.01 M PBS. Tumor volumes were calculated by the formula. 0.52 × a (length) × b (width)^2^ in millimeters. The animals were sacrificed and the tumors were excised after 40 days.

### Immunohistochemistry

In order to quench endogenous peroxidase activities, 4 μm-thick tumor sections were made and incubated with 3% hydrogen peroxide. Heat mediation in citrate buffer (pH 6.0) was used to retrieve antigen. After blocking with 10% goat serum, the slides was incubated with primary antibody. Then, the samples were incubated with the antibodies against Ki-67^48^,^49^ (Abcam) or CD105^9^ (Abcam) overnight in a humidified container at 4 °C. 0.01 M PBS without primary antibodies was applied as the negative control. Immunohistochemical staining was performed with DAB and were counterstained with hematoxylin. Instead of adding antibody, TUNEL reaction mixture (R&D Systems, Minneapolis, MN) was then added to tumor sections, followed by incubation in a humidified chamber at 37 °C for 60 min and DAPI staining for 3 min.

### Statistical analysis

Quantitative data were expressed as mean ± SD. One-way analysis of variance was used to determine significance. The difference was significant when *P* values were < 0.05.

## Additional Information

**How to cite this article:** Huang, Y. *et al*. Fusions of Tumor-derived Endothelial Cells with Dendritic Cells Induces Antitumor Immunity. *Sci. Rep.* **7**, 46544; doi: 10.1038/srep46544 (2017).

**Publisher's note:** Springer Nature remains neutral with regard to jurisdictional claims in published maps and institutional affiliations.
