# Author Correction: Fusions of Tumor-derived Endothelial Cells with Dendritic Cells Induces Antitumor Immunity

**DOI:** 10.1038/s41598-025-12203-3

**Published:** 2025-07-23

**Authors:** Yingying Huang, Qiqi Mao, Jian He, Jing Su, Yi Peng, Wei Liang, Zixi Hu, Sufang Zhou, Xiaoling Lu, Yongxiang Zhao

**Affiliations:** National Center for International Research of Biological Targeting Diagnosis and Therapy, Guangxi Key Laboratory of Biological Targeting Diagnosis and Therapy Research, Collaborative Innovation Center for Targeting Tumor Diagnosis and Therapy, Guangxi Medical University, Shuang Yong Rd. 22, Nanning, 530021 P. R. China

Correction to: *Scientific Reports* 10.1038/srep46544, published online 24 April 2017

This Article contains errors.

As a result of an error during figure assembly, incorrect images are displayed for the H22 Cell Line condition in Figure 1c and for the DC condition in Figure 4c.

The correct figures and accompanying legends appear below.

**Fig. 1:**
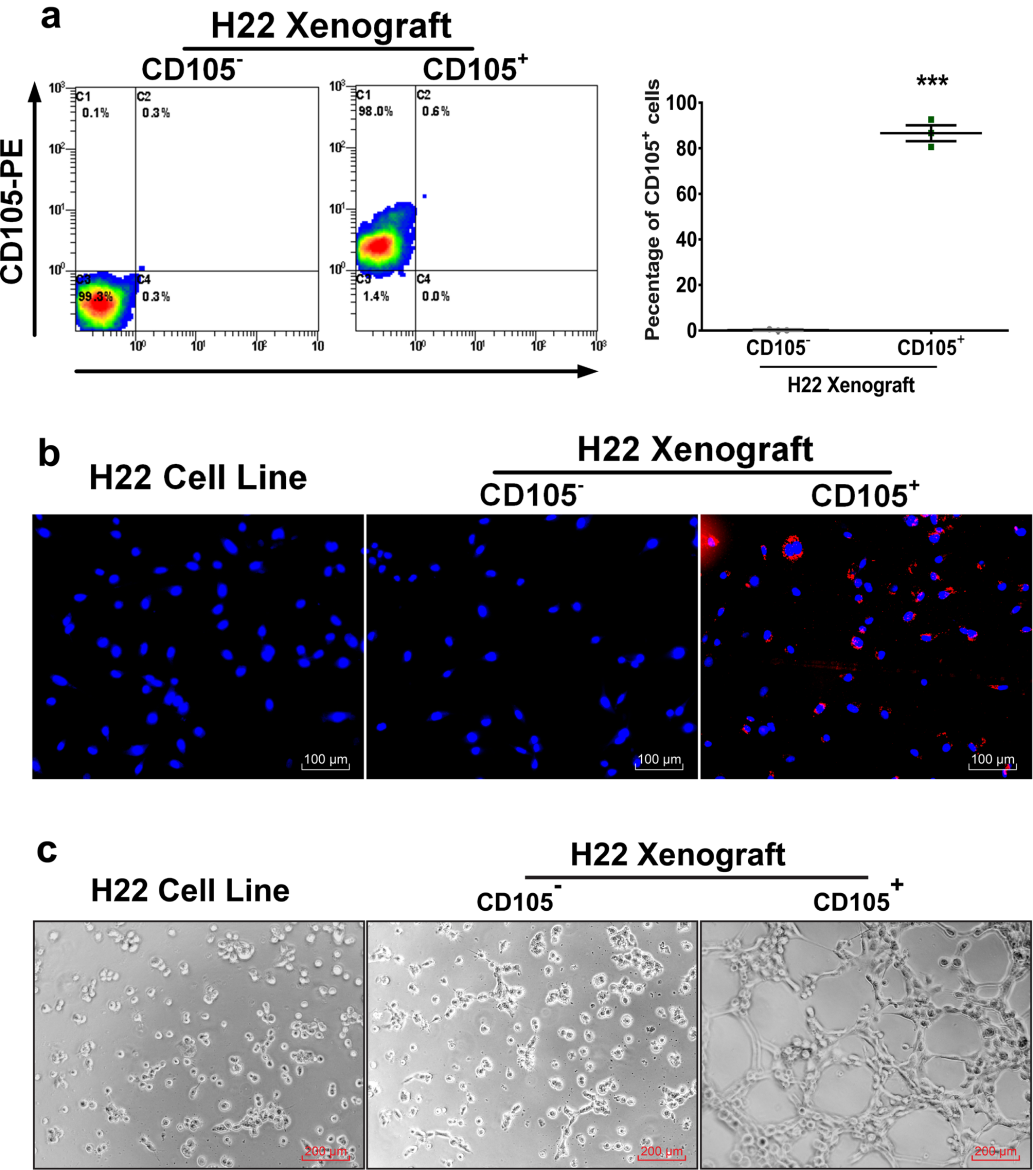
(**a**) Flow cytometry plot data obtained using CD105^+^ antibody to quantify endothelial cells. (**b**) CD105^+^ cells take up acetylated LDL compared to CD105^−^ cells and H22 cell lines. (**c**) CD105^+^ cells form capillary-like networks on Matrigel compared to CD105^−^ cells and H22 cell lines. Scale bars = 100 μm in (**b**), = 200 μm in c. ***P < 0.001.

**Fig. 4:**
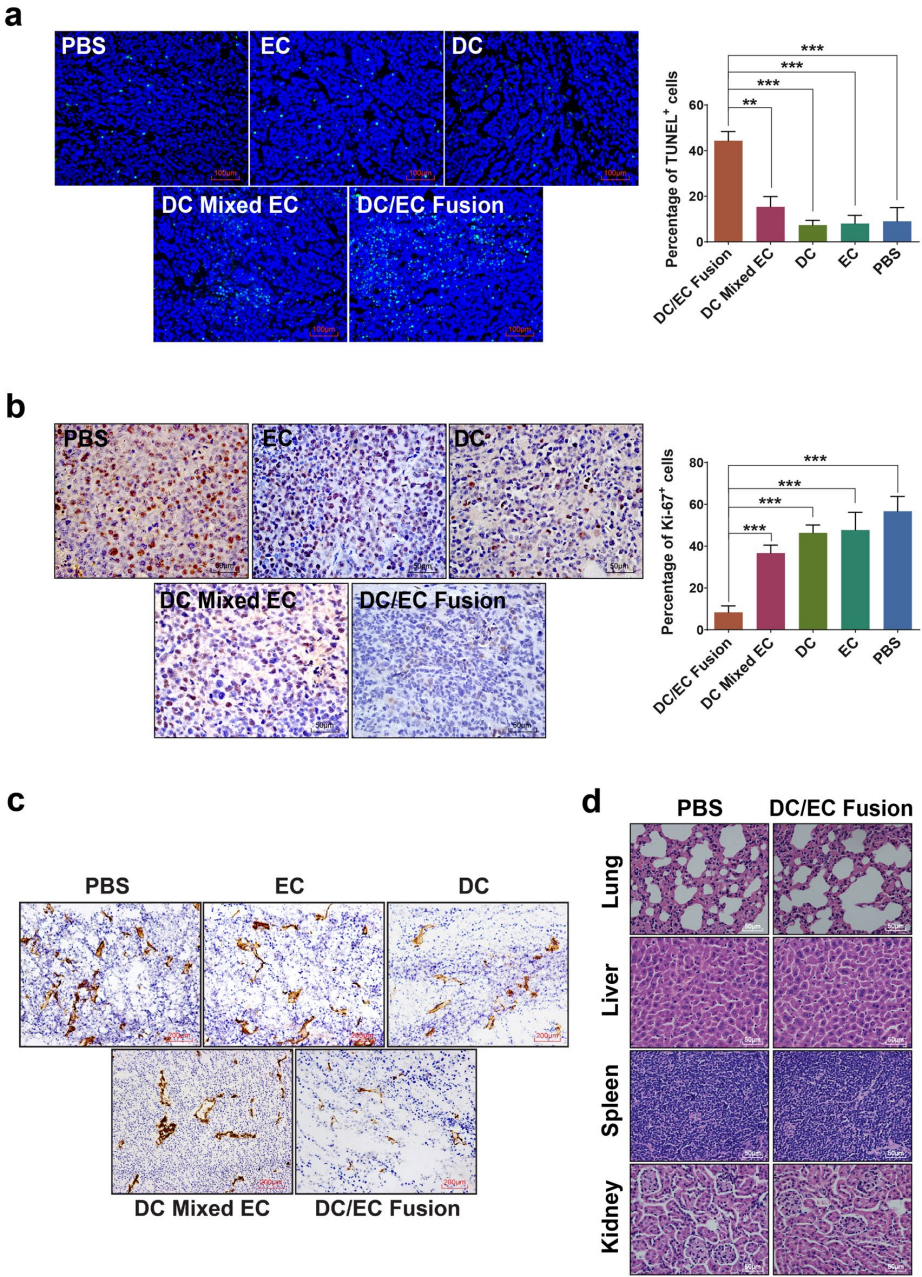
(**a**) TUNEL expression in tumor tissues and quantitation of its expression. (**b**) Ki-67 expression in tumor tissues and quantitation of Ki-67 expression. (**c**) CD105 expression in tumor tissues. (**d**) HE staining of mice organs to determine toxicity of tissues. Scale bars = 100 μm in a, = 50 μm in b and d, = 200 μm in (**c**). **P < 0.01, ***P < 0.001.

